# Author Correction: CircFNDC3B regulates osteoarthritis and oxidative stress by targeting miR-525-5p/HO-1 axis

**DOI:** 10.1038/s42003-023-04863-6

**Published:** 2023-05-11

**Authors:** Zizheng Chen, Yizhen Huang, Yu Chen, Xiaodong Yang, Jinjin Zhu, Guang Xu, Shuying Shen, Ziang Hu, Peihua Shi, Yan Ma, Shunwu Fan

**Affiliations:** 1 Department of Orthopaedic Surgery, Sir Run Run Shaw Hospital, Zhejiang University School of Medicine, 3 East Qingchun Road, Hangzhou, 310016 Zhejiang Province China; 2 Key Laboratory of Musculoskeletal System Degeneration and Regeneration Translational Research of Zhejiang Province, Hangzhou, 310016 Zhejiang Province China; 3 Zhejiang University School of Medicine, Hangzhou, 310016 China

**Keywords:** Mechanisms of disease, Osteoarthritis, RNAi

Correction to: *Communications Biology* 10.1038/s42003-023-04569-9, published online 20 February 2023.

The original version of this Article contained an error in which an incorrect panel **d** of figure 9 was used. In the new version the correct panel for Figure 9 **d** is used.

