# Supplementary material for: CircFNDC3B regulates osteoarthritis and oxidative stress by targeting miR-525-5p/HO-1 axis
Source: Commun Biol. 2023 Feb 20;6:200. doi: 10.1038/s42003-023-04569-9 (PMC9941484; doi:10.1038/s42003-023-04569-9)
Supplement: Supplementary file 2 — Supplementary Materials [file 42003_2023_4569_MOESM2_ESM.pdf]

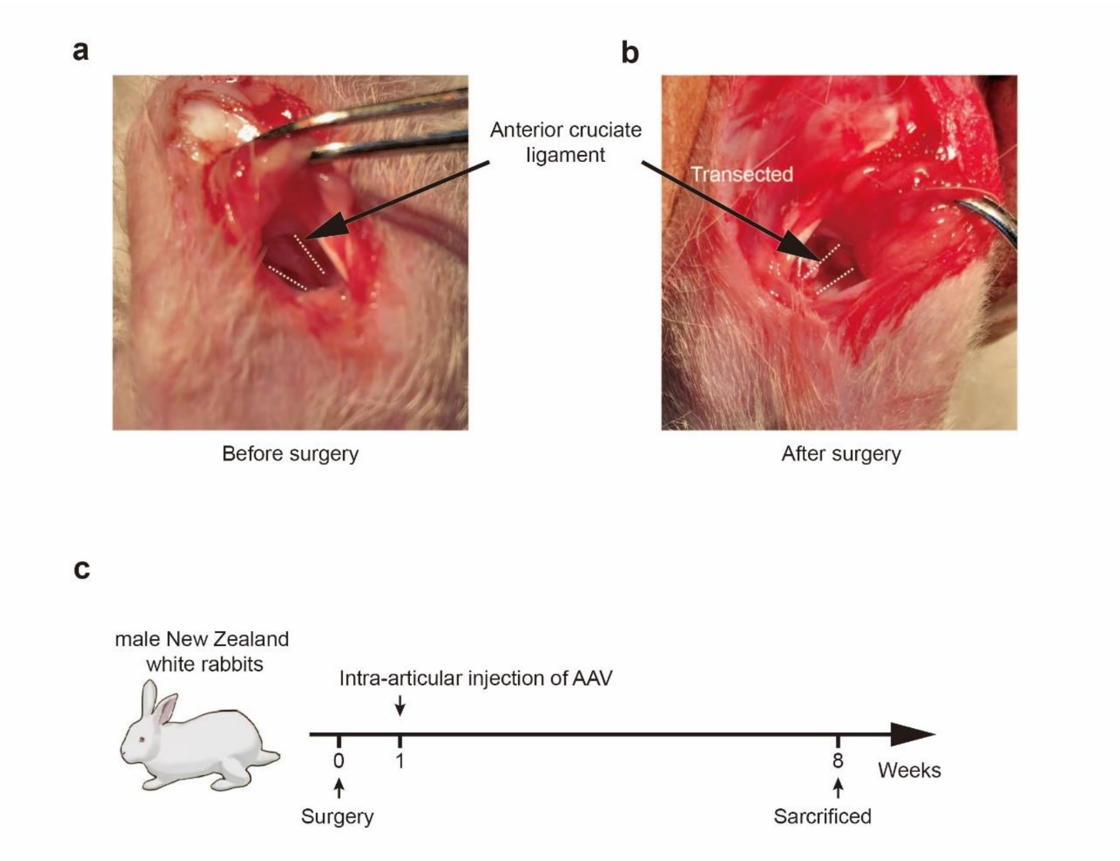

Supplementary Figure S1. **a and b.** Picture of rabbit knee before and after ACLT surgery. **c.** The visualization timeline of animal experiment.

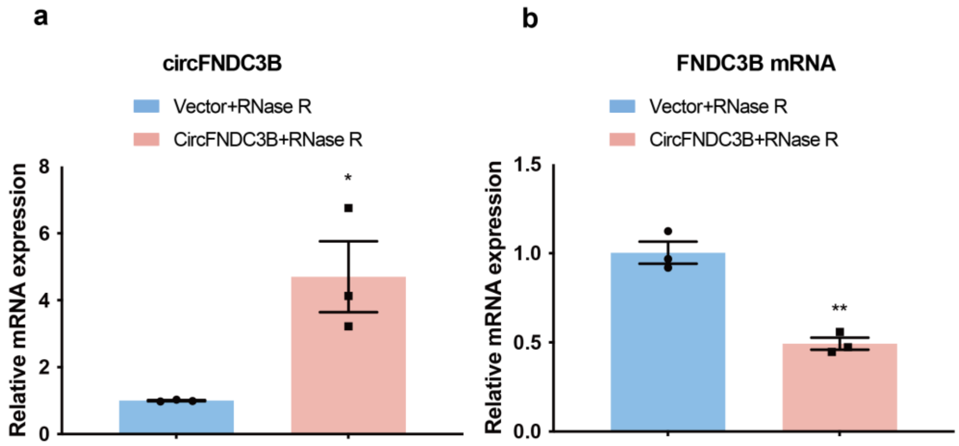

Supplementary Figure S2. RT-qPCR analysis of the relative level of circFNDC3B and FNDC3B mRNA in the HCs after overexpression of CircFNDC3B and treated with RNase R. (n = 3). $^{*}p < 0.05$; $^{**}p < 0.01$ by Student's t test.

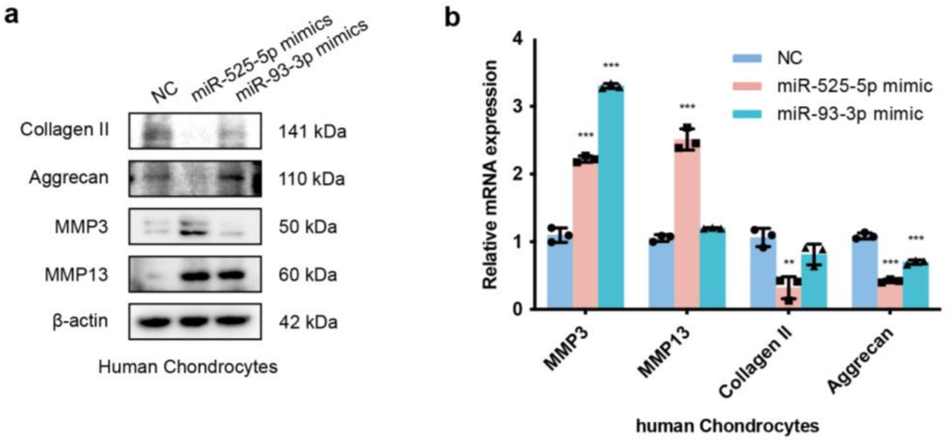

Supplementary Figure S3. WB and RT-qPCR results showed the influence of miR-525-5p and miR-93-3p on the protein expression of matrix-metabolism components. **$\boldsymbol{p}$ < 0.01; ***$\boldsymbol{p}$ < 0.001 by Student's t test.

Supplementary Figure S4

Figure 2c

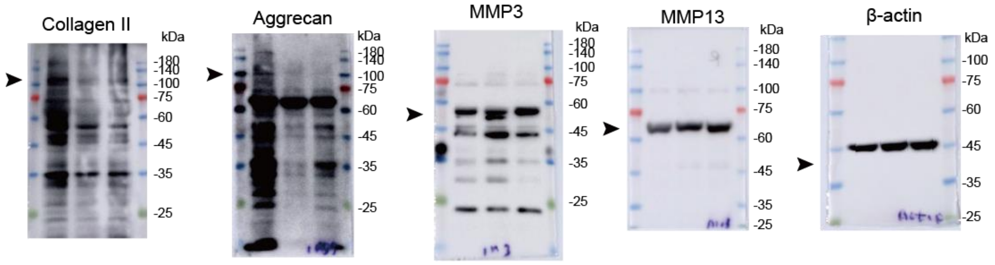

Figure 3c

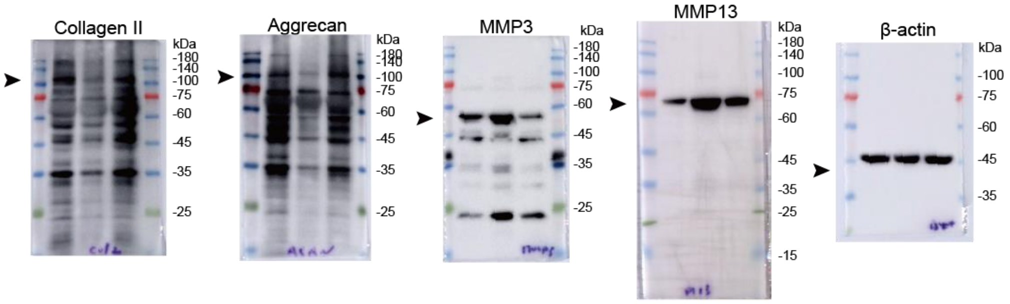

Figure 5c

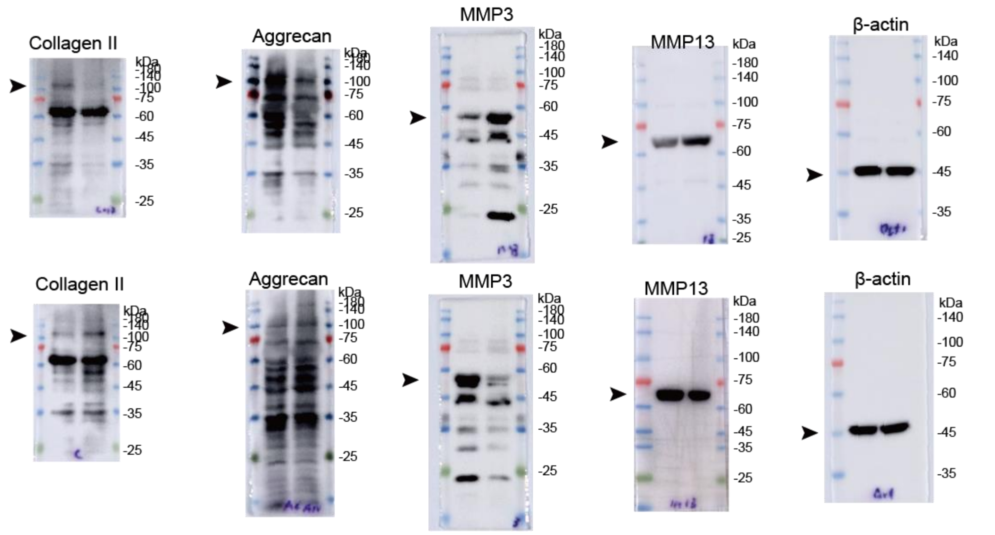

Supplementary Figure S4

Figure 5e

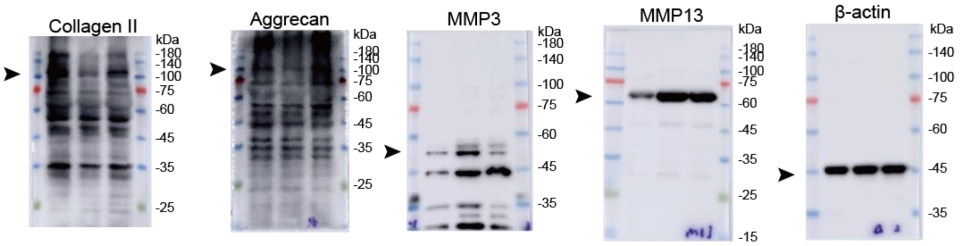

Figure 6e

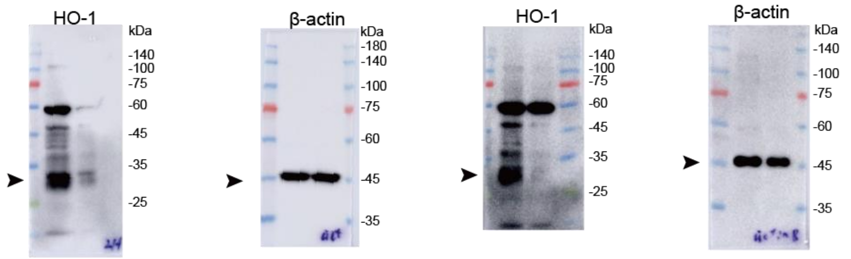

Figure 7c

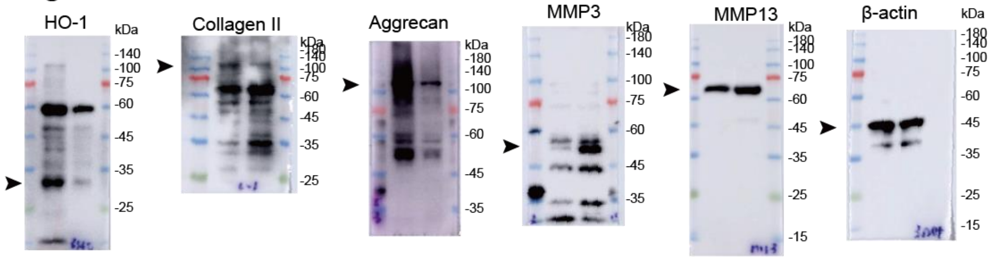

Figure 7e

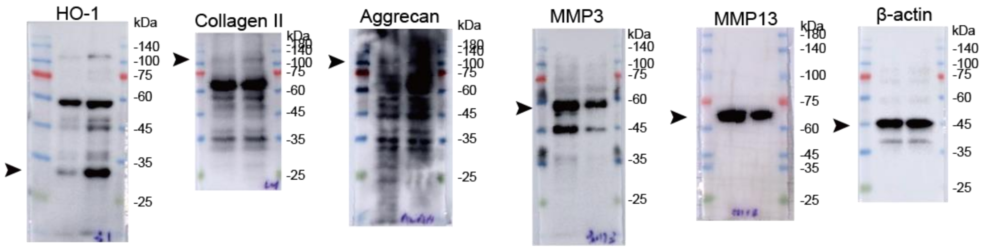

Supplementary Figure S4

Figure 7g

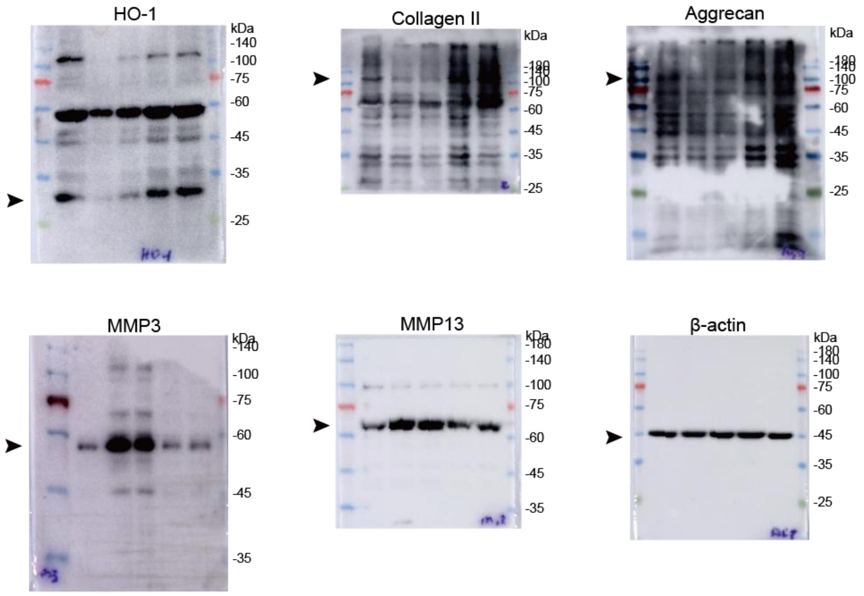

Figure 8b

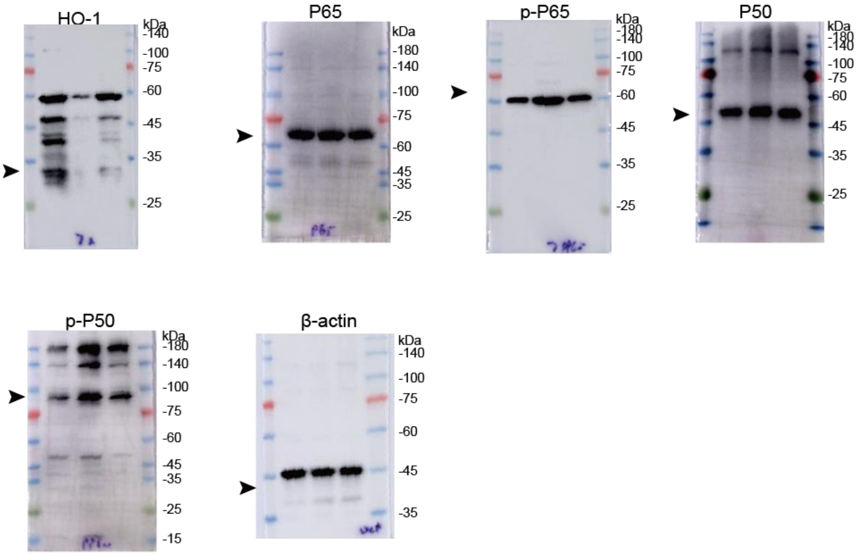

Supplementary Figure S4

Figure 8c

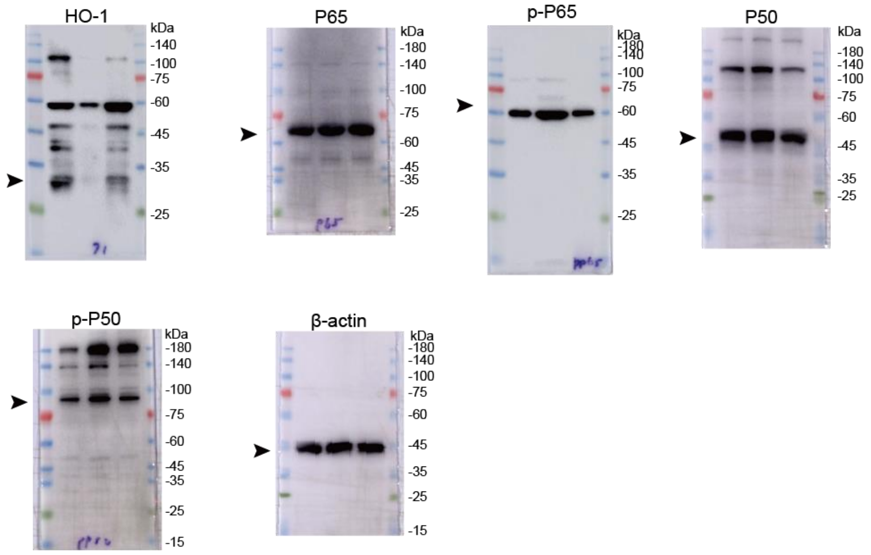

Figure 9e

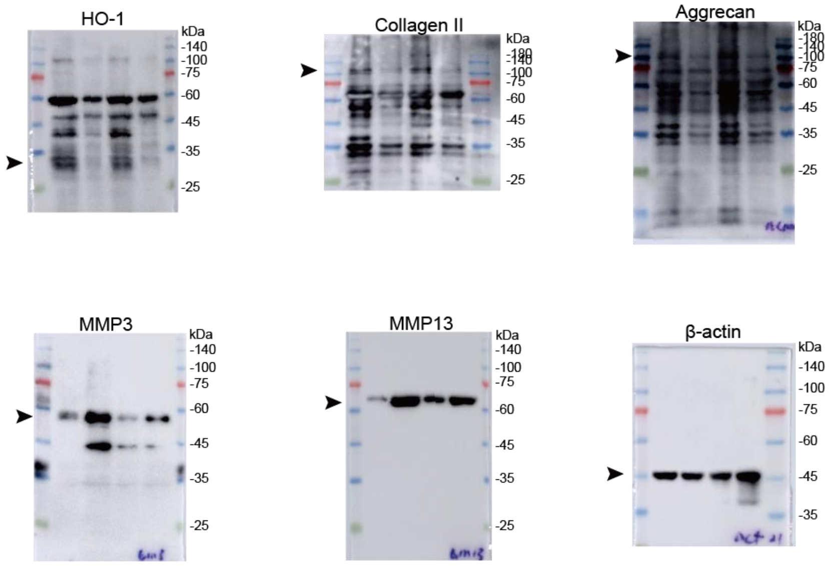

Supplementary Figure S4

Supplementary Figure S3a

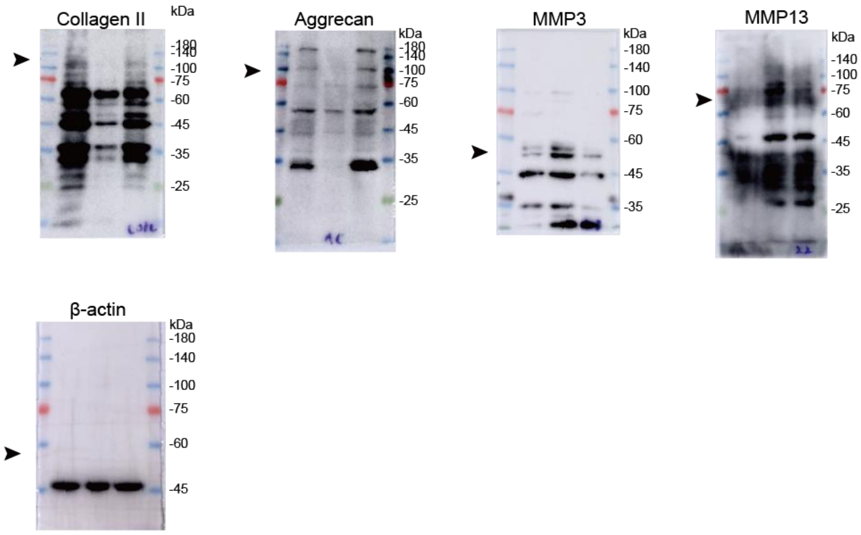

Supplementary Figure S4. Representative uncropped western blot images.

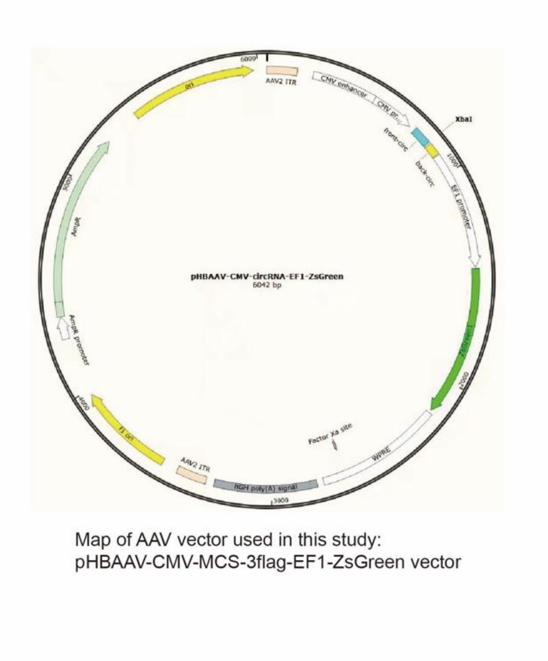

Supplementary Figure S5. Map of AAV vector used in this study.

Supplementary Table S1. Primers used in this study

Gene	Species	Primers
β-actin-F	human	AGAGCTACGAGCTGCCTGAC
β-actin-R	human	AGCACTGTGTTGGCGTACAG
hsa_circFNDC3B-F	human	CAGTGCATTCAAGGAAGCCA
hsa_circFNDC3B-R	human	TTCAGCAATGGTGGCAGTTC
FNDC3B-F	human	CCCATCAAACCCTCCTGGTAG
FNDC3B-R	human	TTCCCTCAACTGGCTTCCTTC
hsa-miR-1296-3p	human	GAGTGGGGCTTCGACCCTAACC
hsa-miR-3151-5p	human	GGTGGGGCAATGGGATCAGGT
hsa-miR-370-3p	human	GCCTGCTGGGGTGGAACCT
hsa-miR-4663	human	AGCTGAGCTCCATGGACGTGCA
hsa-miR-525-5p	human	ccgCTCCAGAGGGATGCACTTTCT
hsa-miR-93-3p	human	cACTGCTGAGCTAGCACTTCCCG
MMP3-F	human	CCTACAAGGAGGCAGGCAAG
MMP3-R	human	CCCGTCACCTCCAATCCAAG
MMP13-F	human	TCGGCCACTCCTTAGGTCTT
MMP13-R	human	AAGTGGCTTTTGCCGGTGTA
COL2A1-F	human	CCAGATGACCTTCCTACGCC
COL2A1-R	human	TTCAGGGCAGTGTACGTGAAC
Aggrecan-F	human	AAGGGCGAGTGGAATGATGT
Aggrecan-R	human	CGTTTGTAGGTGGTGGCTGTG
HO-1-F	human	ACTGCGTTCCTGCTCAACAT
HO-1-R	human	GGGGGCAGAATCTTGCACTTT
PTEN-F	human	TTTTCTTCAGCCACAGGCTC
PTEN-R	human	TGCTTTGAATCCAAAAACCTTACTA
SPP1-F	human	ATCTCCTAGCCCCACAGACC
SPP1-R	human	CACACTATCACCTCGGCCAT
APLN-F	human	TGCTCCTGGCTGTAGTTTGG
APLN-R	human	CCAAATGAAGGTTTGGGGCG
BAX-F	human	CATGGGCTGGACATTGGACT
BAX-R	human	AAAGTAGGAGAGGAGGCCGT
FN1-F	human	ACAAGCATGTCTCTCTGCCA
FN1-R	human	TTTGCATCTTGGTTGGCTGC
TNFRSF1A-F	human	CTGGAGCTGTTGGTGGGAAT
TNFRSF1A-R	human	CTGAGGCAGTGTCTGAGGTG
OLR1-F	human	CCCAGGTGTCTGACCTCCTA
OLR1-R	human	TGCTGGATGAAGTCCTGAACAAT

Supplementary Table S2. Antibodies used in this study

antibody	Prostitution	Place of Origin	Diluted ratio for WB	Diluted ratio for IF	Cat No.
MMP13	abcam	Cambridge, UK	1:1000	1:100	ab51072
MMP3	abcam	Cambridge, UK	1:1000	1:100	ab52915
COL2A1	abcam	Cambridge, UK	1:1000	1:100	ab34712
ACAN	abcam	Cambridge, UK	1:1000	1:100	ab36861
HO-1	abcam	Cambridge, UK	1:1000	Unused	ab52947
p65	CST	Massachusetts, USA	1:1000	Unused	8242
p-p65	CST	Massachusetts, USA	1:1000	Unused	3033
p50	abcam	Cambridge, UK	1:1000	Unused	ab32360
p-p50(S337)	abcam	Cambridge, UK	1:1000	Unused	ab194729
β-actin	CST	Massachusetts, USA	1:2000	Unused	4970s
