# Supplementary material for: CircFNDC3B regulates osteoarthritis and oxidative stress by targeting miR-525-5p/HO-1 axis
Source: Commun Biol. 2023 Feb 20;6:200. doi: 10.1038/s42003-023-04569-9 (PMC9941484; doi:10.1038/s42003-023-04569-9)
Supplement: Supplementary file 5 — Reporting Summary [file 42003_2023_4569_MOESM5_ESM.pdf]

nature portfolio

Corresponding author(s): Shunwu Fan

Last updated by author(s): Sep 20, 2022

Reporting Summary

Nature Portfolio wishes to improve the reproducibility of the work that we publish. This form provides structure for consistency and transparency in reporting. For further information on Nature Portfolio policies, see our Editorial Policies and the Editorial Policy Checklist.

Statistics

For all statistical analyses, confirm that the following items are present in the figure legend, table legend, main text, or Methods section.

n/a	Confirmed	
☐	☒	The exact sample size (*n*) for each experimental group/condition, given as a discrete number and unit of measurement
☐	☒	A statement on whether measurements were taken from distinct samples or whether the same sample was measured repeatedly
☐	☒	The statistical test(s) used AND whether they are one- or two-sided<br>*Only common tests should be described solely by name; describe more complex techniques in the Methods section.*
☐	☒	A description of all covariates tested
☐	☒	A description of any assumptions or corrections, such as tests of normality and adjustment for multiple comparisons
☐	☒	A full description of the statistical parameters including central tendency (e.g. means) or other basic estimates (e.g. regression coefficient) AND variation (e.g. standard deviation) or associated estimates of uncertainty (e.g. confidence intervals)
☐	☒	For null hypothesis testing, the test statistic (e.g. *F*, *t*, *r*) with confidence intervals, effect sizes, degrees of freedom and *P* value noted<br>*Give P values as exact values whenever suitable.*
☒	☐	For Bayesian analysis, information on the choice of priors and Markov chain Monte Carlo settings
☐	☒	For hierarchical and complex designs, identification of the appropriate level for tests and full reporting of outcomes
☒	☐	Estimates of effect sizes (e.g. Cohen's *d*, Pearson's *r*), indicating how they were calculated

Our web collection on statistics for biologists contains articles on many of the points above.

Software and code

Policy information about availability of computer code

Data collection	No software were used.
Data analysis	Statistical analyses were performed using SPSS 22.0. Western blotting analysis was performed using Image Lab Software.

For manuscripts utilizing custom algorithms or software that are central to the research but not yet described in published literature, software must be made available to editors and reviewers. We strongly encourage code deposition in a community repository (e.g. GitHub). See the Nature Portfolio guidelines for submitting code & software for further information.

Data

Policy information about availability of data

All manuscripts must include a data availability statement. This statement should provide the following information, where applicable:

- Accession codes, unique identifiers, or web links for publicly available datasets
- A description of any restrictions on data availability
- For clinical datasets or third party data, please ensure that the statement adheres to our policy

The data that support the findings of this study are openly available from the corresponding author upon reasonable request.

Human research participants

Policy information about studies involving human research participants and Sex and Gender in Research.

Reporting on sex and gender	There are no human research participants involved.
Population characteristics	There are no human research participants involved.
Recruitment	There are no human research participants involved.
Ethics oversight	There are no human research participants involved.

Note that full information on the approval of the study protocol must also be provided in the manuscript.

Field-specific reporting

Please select the one below that is the best fit for your research. If you are not sure, read the appropriate sections before making your selection.

☒ Life sciences ☐ Behavioural & social sciences ☐ Ecological, evolutionary & environmental sciences

For a reference copy of the document with all sections, see nature.com/documents/nr-reporting-summary-flat.pdf

Life sciences study design

All studies must disclose on these points even when the disclosure is negative.

Sample size	The sample sizes of the clinical samples and animal experiment were based on experience and existing literature.
Data exclusions	No data were excluded from the analyses.
Replication	All experiments were run in triplicate to verify the reproducibility of the experimental findings.
Randomization	All 32 rabbits were randomly distributed into 4 groups of 8 rabbits per group.
Blinding	Cartilage destruction was scored by observers blinded to group-identifying information using the OARSI grading system.

Reporting for specific materials, systems and methods

We require information from authors about some types of materials, experimental systems and methods used in many studies. Here, indicate whether each material, system or method listed is relevant to your study. If you are not sure if a list item applies to your research, read the appropriate section before selecting a response.

Materials & experimental systems

n/a	Involved in the study	
☐	☒	Antibodies
☒	☐	Eukaryotic cell lines
☒	☐	Palaeontology and archaeology
☐	☒	Animals and other organisms
☒	☐	Clinical data
☒	☐	Dual use research of concern

Methods

n/a	Involved in the study	
☒	☐	ChIP-seq
☐	☒	Flow cytometry
☒	☐	MRI-based neuroimaging

Antibodies

Antibodies used

MMP13; abcam; Cambridge, UK; ab51072
MMP3; abcam; Cambridge, UK; ab52915
ADAMTS5; abcam; Cambridge, UK; ab41037
COL2A1; abcam; Cambridge, UK; ab34712
ACAN; abcam; Cambridge, UK; ab3778
HO-1; abcam; Cambridge, UK; EPR1390Y
p65; CST; Massachusetts, USA; 8242
p-p65; CST; Massachusetts, USA; 3033

p50; abcam; Cambridge, UK; ab32360
p-p50(S337); abcam; Cambridge, UK; ab194729
β-actin; CST; Massachusetts, USA; 4970s

Validation

MMP13: The Abpromise guarantee covers the use of ab51072 in the WB application. Abcam species reactivity states that ab51072 reacts with Human. Manufacturer's website: https://www.abcam.com/mmp13-antibody-ep1263y-ab51072.html
MMP3: The Abpromise guarantee covers the use of ab52915 in the WB, IHC-P, ICC/IF applications. Abcam species reactivity states that ab52915 reacts with Mouse, Rat, Human, Recombinant fragment. Manufacturer's website: https://www.abcam.com/mmp3-antibody-ep1186y-ab52915.html
ADAMTS5: The Abpromise guarantee covers the use of ab231595 in the IHC-P, WB applications. Abcam species reactivity states that ab231595 reacts with Human, Recombinant fragment. Manufacturer's website: https://www.abcam.cn/adamts5-antibody-ab231595.html
Collagen II: The Abpromise guarantee covers the use of ab188570 in the WB application. Abcam species reactivity states that ab188570 reacts with Rat, Human. Manufacturer's website: https://www.abcam.cn/collagen-ii-antibody-epr12268-ab188570.html
Aggrecan: The Abpromise guarantee covers the use of ab3778 in the WB, IHC-P applications. Abcam species reactivity states that ab3778 reacts with Human, Recombinant fragment. Manufacturer's website: https://www.abcam.cn/aggrecan-antibody-6-b-4-ab3778.html
HO-1: The Abpromise guarantee covers the use of ab52947 in the Flow Cyt (Intra), WB, IHC-P, IP applications. Abcam species reactivity states that ab52947 reacts with Mouse, Human. Manufacturer's website: https://www.abcam.cn/heme-oxygenase-1-antibody-ep1391y-ab52947.html
p65: The Cell Signaling Technology guarantee covers the use of product 8242 in the WB, IP, IHC, IF, Flow Cytometry, ChIP applications. CST species reactivity states that product 8242 reacts with Human, Mouse, Rat, Hamster, Monkey, Dog. Manufacturer's website: https://www.cellsignal.com/products/primary-antibodies/nf-kb-p65-d14e12-xp-rabbit-mab/8242?site-search-type=Products&N=4294956287&Ntt=p65&fromPage=plp
p-p65: The Cell Signaling Technology guarantee covers the use of product 3033 in the WB, IP, IF, Flow Cytometry applications. CST species reactivity states that product 3033 reacts with Human, Mouse, Rat, Hamster, Monkey, Pig. Manufacturer's website: https://www.cellsignal.cn/products/primary-antibodies/phospho-nf-kb-p65-ser536-93h1-rabbit-mab/3033?site-search-type=Products&N=4294956287&Ntt=p65&fromPage=plp
β-actin: The Cell Signaling Technology guarantee covers the use of product 3700 in the WB, IHC, IF, Flow Cytometry applications. CST species reactivity states that product 3700 reacts with Human, Mouse, Rat, Hamster, Monkey, Dog. Manufacturer's website: https://www.cellsignal.cn/products/primary-antibodies/b-actin-8h10d10-mouse-mab/3700?site-search-

Animals and other research organisms

Policy information about studies involving animals; ARRIVE guidelines recommended for reporting animal research, and Sex and Gender in Research

Laboratory animals

All rabbits (12-month-old male New Zealand white rabbits, weighing 2.5-3 kg) were purchased from Xin Jian rabbit field (Certificate No. SCXK, Zhejiang, 2015-0004, China).

Wild animals

No wild animals were involved in the study.

Reporting on sex

We respect every gender. Sex is not considered in the study.

Field-collected samples

No samples were collected from the field in the study.

Ethics oversight

Collections of human cartilage samples were according to protocols approved by the Ethics Committee of Sir Run Run Shaw Hospital (Hangzhou, China), and the methods were carried out in accordance with the approved guidelines.
The animal experiments were performed strictly with the approval of the Institute of Health Sciences Institutional Animal Care and Use Committee (Zhejiang, China).

Note that full information on the approval of the study protocol must also be provided in the manuscript.

Flow Cytometry

Plots

Confirm that:

☒ The axis labels state the marker and fluorochrome used (e.g. CD4-FITC).

☒ The axis scales are clearly visible. Include numbers along axes only for bottom left plot of group (a 'group' is an analysis of identical markers).

☒ All plots are contour plots with outliers or pseudocolor plots.

☒ A numerical value for number of cells or percentage (with statistics) is provided.

Methodology

Sample preparation

The Beyotime Biotech (China) ROS assay kit was used to determine the levels of intracellular ROS. Chondrocytes (biological source: human) receiving different treatments were collected using trypsin (Thermo Fisher Scientific) and washed twice with PBS. Then the chondrocytes were centrifuged, and the supernatant was discarded. Finally, the chondrocytes were incubated with DCFH-DA (10 mmol/L) at 37℃ for 30 min in a darkroom for analysis by flow cytometry.

Instrument	BD FACS Calibur flow cytometer (BD Biosciences).
Software	BD FACStation™ Software
Cell population abundance	The cell population abundance is 10000 or 20000 cells per sample.
Gating strategy	Gating strategy that involved in this study are openly available from the corresponding author upon any request. If needed, we will add the gating strategy figures in supplementary materials.

☐ Tick this box to confirm that a figure exemplifying the gating strategy is provided in the Supplementary Information.
